# Supplementary material for: Field-based molecular detection of Batrachochytrium dendrobatidis in critically endangered Atelopus toads and aquatic habitats in Ecuador
Source: PLoS One. 2024 Mar 14;19(3):e0299246. doi: 10.1371/journal.pone.0299246 (PMC10939218; doi:10.1371/journal.pone.0299246)
Supplement: S1 File — (DOCX) [file pone.0299246.s001.docx]

**Supplementary Material SM1.**

Standardized protocols to extract environmental DNA (eDNA) from water samples, DNA from SWABs, and amplifying PCR products to detect Bd with the field laboratory

- - - 1. **Protocol for sterilizing laboratory equipment for eDNA analysis**

**Materials:**

- 1 L plastic bottles for eDNA water samples
- Glass vacuum filtration system
- Petri dishes
- Aluminum foil
- PORAFIL™ nitrocellulose membrane filters, Pore Size 0.45 µm, Diameter 47 mm

**NOTE:** The number of materials required depends on the number of sites planned for fieldwork. For each site 1 bottle of MilliQ water is transported as a control, and 1 bottle of 1L water are collected for eDNA analysis

**Reagents:**

- Drinking water
- Detergent
- Sodium hypochlorite 1%.
- 70% ethanol
- MilliQ water
- **Sterile distilled water**

**NOTE:** MiliQ water (distilled and deionized water, Type I) is used as a control during sample transport.

**Equipment:**

- Autoclave
- Stove

**Procedure:**

**Sterilization of bottles at laboratory**

- 1. Wash and rinse the bottles with potable water and detergent.
  2. Remove any residual DNA and amplicons from the bottles with 1% sodium hypochlorite, shaking for 5 min. Rinse with sterile distilled water
  3. Clean the bottles with 70% ethanol, shaking for 2 min.
  4. Sterilize the bottles at 121ºC for 30 min and 1.2 bar of pressure.
  5. Cool the bottles to room temperature and cap them for storage.

**Sterilization of filtration system**

- - - 1. Wash and rinse the glass filtration system and Petri dishes with potable water and detergent.

1. Clean each piece with 1% sodium hypochlorite and 70% ethanol .
2. Dry and wrap all the pieces in aluminum foil for sterilizing at 121ºC for 30 min and 1.2 bar of pressure.
3. Oven-dry all the pieces.

**Membrane sterilization**

1. If the membrane is not sterilized and not individually package, sterilize the membrane filters at 121ºC for 30 min and 1.2 bar .
2. **Protocol for water collection and sample filtration**

**Materials:**

- 1 L plastic bottles of collected water for eDNA.
- Glass vacuum filtration system
- Petri dishes
- PORAFIL™ nitrocellulose membrane filters, Pore Size 0.45 µm, Diameter 47 mm
- Alcohol lamp
- Nitrile gloves
- Mouthpiece or mask
- Lab coat

**Reagents:**

- 70% Ethanol

**Procedure:**

- - - - 1. Collect 1 litre of water, ensuring that about 350 ml is spurced from a distinct point whithin the water body. For instance, in a small river distributed the three sampling points along the lenght of the river. In contrast, when sampling a large river, collect water from three points aligned transversely across the river – one from each bank and one from the center. In the case of a lake, if it is large and a boat is available, samples should be taken from the edges and the center of the lake.
  1. n the laboratory, filter the litre of MillliQ water transported as a control on a single membrane filter.
  2. After filtering, remove the filter from the system and store in a sterilized petri dish at -20°C until used for DNA extraction.
  3. For the collected water samples, filter the 1L of water collected per sampling site.
  4. Using sterile forceps, remove the membrane filter from the filtration system and store it in a sterilized petri dish
  5. Cut filters in half. Use one half for DNA extraction in the field with portable devices, and store the other half at -20 °C for subsequent extraction in the university laboratory. Cut each half of the filter into small strips, 1 to 2 mm in size. Handle these strips with sterilized tools and store them in sterile 1.5 ml Eppendorf tubes.
  6. Use a sterilize filtration system or desinfected the filtration system between samples to avoid cross contamination

1. **Protocol for DNA extraction from water fiters**

This protocol has been adapted from Gebaseerd op Asselman (2006) in Sambrook et al. (1989), and a version of the Epicenter Masterpure DNA Purification Kit used by Riascos *et al.* [34] for field samples.

**Materials:**

- Nitrile gloves
- Face mask
- Lab coat
- Set of micropipettes
- Alcohol lamp
- Scalpel and dissecting forceps
- Sterile 1.5 ml Eppendorf tubes
- Sterile pistil for Eppendorf tube
- Ice or cold block
- Rack for Eppendorf tubes

**Reagents:**

- 70% ethanol
- Extraction buffer: 10mMTris-Cl (pH 8.0); 0.1M EDTA (pH 8.0); 0.5% SDS. (sterilized)
- Precipitation buffer: 5M ammonium acetate (sterile).
- Proteinase K ( K 50 µg/µL)
- RNAse (4 mg/mL)
- Isopropanol
- MillliQ water (sterilized).

**Equipment:**

- Vortex
- Portable thermoblock
- Portable centrifuge

**Procedure:**

1. Disinfect the workspace with 70% ethanol and turn on the alcohol lamp to sterilize the area and the air. Wear nitrile gloves, mask and lab coat.

At each eppendorf tube with strips of the membrane ,add 300 µL of extraction buffer (sterile) and homogenize using a sterile pistil. Use a new pistil in the homogenization of each sample.

1. Add 300 µL of additional extraction buffer
2. Add two sterile glass beads and vortex for 1 min at maximum speed to homogenize the sample.
3. Add 10 µL of Proteinase K ( K 50 µg/µL), vortex for 20 seconds and incubate at 65 °C for 30 minutes.Mixing by vortex every 10 minutes.
4. Cool the samples in a cold block or container with ice and keep them cold during the entire DNA extraction procedure.
5. Add 175 µL 5M ammonium acetate (sterile) to each sample and vortex at maximum speed for 10 seconds to mix the reagent evenly with the sample solution and membrane.
6. Centrifuge at 13,000 rpm for 10 minutes. Use ice blocks around the centrifuge to prevent overheating.
7. Label a new sterile 1.5 ml Eppendorf tube with the sample code.
8. Transfer as much supernatant volume as possible without removing the pellet to each new pre-labeled tube. Discard the pellet
9. Add 3 µL RNAse (4 mg/mL) to each sample and homogenize by pipetting.
10. Mix the sample by inverting the Eppendorf tube 25 times and incubate at 37°C for 10 minutes.
11. Cool the samples for 5 minutes.
12. Label a new sterile 1.5 ml Eppendorf tube with the sample code.
13. Centrifuge at 12,000 rpm for 10 minutes. Use ice blocks around the centrifuge to prevent overheating.
14. Collect the supernatant with a micropipette, transfer it to the new 1.5ml Eppendorf tube previously labeled and discard the pellet.
15. Add 500 µL of Isopropanol to each sample and mix by inverting the Eppendorf tube for 30-40 times.
16. Centrifuge at 12,000 rpm for 10 minutes. Use ice blocks around the centrifuge to prevent overheating.
17. Discard the Isopropanol in the organic debris taking care not to lose the pellet at the bottom of the Eppendorf tube.
18. Add 500 µL of 70% ethanol and invert the Eppendorf tube several times to recover the pellet.
19. Centrifuge at 12,000 rpm for 3 minutes. Use ice blocks around the centrifuge to prevent overheating.
20. Discard the ethanol in the organic waste, taking care not to lose the pellet.
21. Suspend the opened Eppendorf tubes on a paper towel until the ethanol has evaporated. To accelerate evaporation, it is possible to incubate the samples with the lids open at 65°C for 2-3 minutes. .
22. Suspend the pellet with 40 µL of MillliQ water (sterile).
23. Vortex to homogenize the sample.
24. Store the DNA obtained at -20°C.
25. **Protocol for swab processing and DNA isolation for *Batrachochytrium dendrobatidis* (Bd) detection"**

**Biological sample: cotton swab in lysis buffer**

For the extraction of DNA from the swab samples of the amphibian skin, the *Wizard Genomic DNA Purification Kit* with some technical modifications linked to the type of samples and the laboratory conditions was used.

**Materials:**

- 1.5ml microtubes
- Micropipettes
- pipette tips
- pestles
- racks
- ice
- wash bottles
- gloves
- permanent marker
- Parafilm

**Reagents:**

- Ethanol absolute
- Ethanol 70 %
- Isopropanol
- lysis buffer: Tris-HCl 0.18 M; EDTA 10 mM; SDS 1%, pH 8.2
- Proteinase K 50 µg/µL or 20 µg/µL
- Protein Precipitation Solution
- sodium acetate 3 M pH 5.2
- RNase 4 mg/mL
- DNA Rehydration Solution

**Equipment:**

- Vortex BIOSAN model V-1 Plus
- Microcentrifuge MiniPCR GyroTM
- Thermoblock BIOSAN model Bio TDB-100 or water bath

**Procedure:**

- - - 1. Mix the swabs in 300 µL of the lysis buffer (Tris-HCl 0.18 M; EDTA 10 mM; SDS 1%, pH 8.2).
      2. Remove the swabs using sterile tweezers.
      3. Add 10µL of Proteinase K (50µg/µL) (Invitrogen Cat. # QS0511) and mix the samples.
      4. Vortex the tubes at maximum speed (Vortex V-1 plus) for 20 seconds.
      5. Incubate the tubes on a thermoblock at 65°C for 30 minutes.
      6. Add 2.5 µL of RNase (10 mg/mL) (Thermo Scientific, Cat. # EN0531) and mix the solution by pipetting before incubating at 37°C for 10 minutes.
      7. Add 100µL of protein precipitation solution (Promega) to the samples and vortex for 20 seconds.
      8. Incubate the samples on ice for 10 minutes for cells and protein precipitation.
      9. Centrifuge the samples at 12,000 rpm for 5 minutes.
      10. Recover the supernatant and transfer it to a clean, sterilized 1.5 mL microtube.
      11. Add 40 µL of Sodium acetate (3M, pH 5.2) and 1000µL of absolute ethanol to the supernatant.
      12. Centrifuge the samples for 5 minutes at 12,000 rpm.
      13. Discard the supernatant and remove any remaining droplets with a clean, sterile micropipette tip after spinning.
      14. Dry the microtube in a thermoblock at 60°C until no liquid residue is observed.
      15. Finally, resuspend the DNA in 25 µL of sterile MilliQ water. Alternatively, rehydrate the DNA by incubating the solution overnight at room temperature or 4°C.
      16. Store the DNA at 2-20°C.

1. **Polymerase Chain Reaction (PCR) protocol for swab and water sample analysis**

The primer sequences used are

ITS1-3 Chytr-F 5´ CCTTGATATAATACAGTGTGCCATATGTC 3´ and

5.8S Chytr-R 5´ AGCCAAGAGATCCGTTGTCAA 3´.

**Materials:**

- 0.2 ml microtubes
- Micropipettes
- Sterile filter tips
- racks
- ice
- gloves
- permanent marker

**Chemicals:**

- sterile MillliQ H_2_O
- TaqMan Environmental Master Mix 2.0
- Primer ITS1-3 Chytr-F
- Primer 5.8S Chytr-R

**Equipment:**

- Thermocycler MiniPCR
- Vortex
- Centrifuge

**Software:**

- miniPCR App v2.0 for the cell phone

**Procedure:**

**Preparing the PCR-mix**

1. Disinfect the working zone with ethanol 70%.
2. Insert all the equipment, materials and chemicals in the safety box, lined with fresh aluminum foil, except the DNA to be amplified.
3. Prepare one master mix solution using a 1.5 mL tube with the following components and respective quantities as recommended by the kit. Add the solutions in the following order:

| **Reagent** | **1 RX** |
| --- | --- |
| **Sterile MillliQ water** | 7.5μL |
| **Primer F 10μM** | 2.5μL |
| **Primer R 10μM** | 2.5µL |
| **TaqMan Environmental Master Mix 2.0** | 1uL |
| **DNA** | 1.0μL |
| **Final volume** | 25 μL |

**1 RX**: quantities for 1 run

1. Keep the master Mix cool by using a cooling block
2. Transfer 22 µL master Mix to a microtube of 0.2 mL and store on ice (or in a cooling block)
3. Add 3 µL DNA of each sample to each 0.2 ml tube.
4. Vortex shortly and perform a short-spin for each sample
5. Bring the simples to the miniPCR thermocycler and adjust the amplification conditions in the miniPCR App v2.0 for cell phones

**Amplification conditions**

The following thermal profile for DNA amplification has to be added in the application for cell phones, connected to the miniPCR thermocycler through Bluetooth.

|  | **Temperature** | **Time** |
| --- | --- | --- |
| **Initial denaturation** | 95 °C | 180 seconds |
| **Denaturation** | 98 °C | 20 seconds (35x) |
| **Annealing** | 60 °C | 15 seconds (35x) |
| **Extension** | 72 °C | 15 seconds (35x) |
| **Final extension** | 72°C | 300 seconds |

1. **Protocol for electrophoresis visualization**

**Materials:**

- micropipettes
- pipette tips
- Parafilm
- rack
- gloves
- permanent marker
- paper towel
- Agarose, PCR-grade

**Chemicals:**

- Tris-Borato-EDTA (TBE) 1X
- BlueJuice™ Gel Loading Buffer
- Ladder DNA 100 bp
- PCR products
- SYBR Safe DNA Gel Stain

**Equipment:**

- blueGel™ electrophoresis with built-in trans-illuminator

**Procedure:**

1. The mold has the capacity for preparing two gels simultaneously, enabling the preparation of a single solution. The voltage conditions cannot be changed in the blueGel™ electrophoresis with built-in trans-illuminator
2. Mix 0.3 g of agarose with 15 mL of TBE buffer
3. Heat the solution till lumps are no longer observed
4. Add 1 µL of SYBR Safe for each 10 mL of TBE. In this case, 1.5 µL should be added
5. Pour the solution into the mold and insert the combs
6. Cover the mold with the electrophoresis viewer till the gel has solidified for preventing the degradation of the SYBR Safe.
7. Assemble the electrophoresis chamber and install the solidified gel
8. Add TBE 1X in the electrophoresis chamber till the gel is completely submerged
9. Add 1 µL Loading Dye to 5 µL of PCR product, and keep a registry of the location of the positive and negative control samples.
10. Load 5 µL of each sample in the wells
11. Load 4 µL of the molecular marker (Ladder DNA 100bp) in the first well
12. Cover the chamber and start the equipment
13. Remove the condensation before observing and photographing the gel
