# Supplementary material for: Field-based molecular detection of Batrachochytrium dendrobatidis in critically endangered Atelopus toads and aquatic habitats in Ecuador
Source: PLoS One. 2024 Mar 14;19(3):e0299246. doi: 10.1371/journal.pone.0299246 (PMC10939218; doi:10.1371/journal.pone.0299246)
Supplement: S2 File — See S1 Table for specimens, access numbers in Genbank and water sample codes. (DOCX) [file pone.0299246.s002.docx]

**Supplementary Material SM2.** Sequences (.fasta) of positive Bd validated in BLASTn from SWABs (2395, 2397, 2420, 2421) and water (1a-16a) samples in the study area. See Table S1 for specimens and water sample codes.

>2395_CHYTR.ab1

AATTTATTTATTTTTTCGACAAATTAATTGGAAATTGAATAATTTTATTTTAATTGATTAATAAATATTAAAAACAACTTTCTGACAACGGATCT

>2397_CHYTR.ab1

AATTTATTTATTTTTTCGACAAATTAATTGGAAATTGAATAATTTTATTTTAATTGATTAATAAATATTAAAAACAACTTTCTGACAACGGATCT

>2420_CHYTR.ab1

AATTTATTTATTTTTTCGACAAATTAATTGGAAATTGAATAATTTTATTTTAATTGATTAATAAATATTAAAAACAACTTTCTGACAACGGATCT

>2421_CHYTR.ab1

AATTTATTTATTTTTTCGACAAATTAATTGGAAATTGAATAATTTTATTTTAATTGATTAATAAATATTAAAAACAACTTTCTGACAACGGATCT

>1a_CHYTR.ab1

AATTTATTTATTTTTTCGACAAATTAATTGGAAATTGAATAATTTTATTTTAATTGATTAATAAATATTAAAAACAACTTTCTGACAACGGATCT

>2a_CHYTR.ab1

AATTTATTTATTTTTTCGACAAATTAATTGGAAATTGAATAATTTTATTTTAATTGATTAATAAATATTAAAAACAACTTTCTGACAACGGATCT

>3a_CHYTR.ab1

AATTTATTTATTTTTTCGACAAATTAATTGGAAATTGAATAATTTTATTTTAATTGATTAATAAATATTAAAAACAACTTTCTGACAACGGATCT

>4a_CHYTR.ab1

AATTTATTTATTTTTTCGACAAATTAATTGGAAATTGAATAATTTTATTTTAATTGATTAATAAATATTAAAAACAACTTTCTGACAACGGATCT

>6a_CHYTR.ab1

AATTTATTTATTTTTTCGACAAATTAATTGGAAATTGAATAATTTTATTTTAATTGATTAATAAATATTAAAAACAACTTTCTGACAACGGATCT

>6b_CHYTR.ab1

AATTTATTTATTTTTTCGACAAATTAATTGGAAATTGAATAATTTTATTTTAATTGATTAATAAATATTAAAAACAACTTTCTGACAACGGATCT

>7a_CHYTR.ab1

AATTTATTTATTTTTTCGACAAATTAATTGGAAATTGAATAATTTTATTTTAATTGATTAATAAATATTAAAAACAACTTTCTGACAACGGATCT

>16a_CHYTR.ab1

AATTTATTTATTTTTTCGACAAATTAATTGGAAATTGAATAATTTTATTTTAATTGATTAATAAATATTAAAAACAACTTTCTGACAACGGATC
