# Supplementary material for: Field-based molecular detection of Batrachochytrium dendrobatidis in critically endangered Atelopus toads and aquatic habitats in Ecuador
Source: PLoS One. 2024 Mar 14;19(3):e0299246. doi: 10.1371/journal.pone.0299246 (PMC10939218; doi:10.1371/journal.pone.0299246)
Supplement: S2 Table — (DOCX) [file pone.0299246.s004.docx]

**Table S2.** Literature review of amphibians with detected Bd in Ecuador. ND = No data available.

| **Taxonomy** | **N** | **Positive for Bd** | **Site** | **Province** | **GPS coordinates** | **Altitude (m asl)** | **Bibliography** |
| --- | --- | --- | --- | --- | --- | --- | --- |
| **Family: Bufonidae** |  |  |  |  |  |  |  |
| *Atelopus bomolochos* | ND | ND | ND | Carchi y Azuay | ND | 3100-4000 | [1] |
| *Atelopus ignescens* |  |  | Páramo de Guamaní | ND | 78.19969°O, 0.34627°S | ND | [2] |
| *Atelopus nanay* | 14 | 1 | CCA-Amaru | Azuay | ND | ND | [3] |
| *Atelopus sp.* | 26 | 8 | Sureste del Ecuador | ND | ND | 1100 | [4] |
| **Family: Centrolenidae** |  |  |  |  |  |  |  |
| *Centrolene ballux* | 26 | 17 | RLG | Pichincha | 0°01’S, 78°44’W | 1822-2400 | [5] |
| *Centrolene heloderma* | 7 | 1 | RLG | Pichincha | 0°01’S, 78°44’W | 1822-2400 | [5] |
| *Centrolene lynchi* | 11 | 2 | RLG | Pichincha | 0°01’S, 78°44’W | 1822-2400 | [5] |
| *Centrolene peristictum* | 37 | 9 | RLG | Pichincha | 0°01’S, 78°44’W | 1822-2400 | [5] |
| *Nymphargus grandisonae* | 21 | 5 | RLG | Pichincha | 0°01’S, 78°44’W | 1822-2400 | [5] |
| *Nymphargus griffithsi* | 5 | 0 | RLG | Pichincha | 0°01’S, 78°44’W | 1822-2400 | [5] |
| *Nymphargus lasgralarias* | 26 | 8 | RLG | Pichincha | 0°01’S, 78°44’W | 1822-2400 | [5] |
| *Teratohyla midas* | 4 | 0 | Wisui-Cutucú | Morona Santiago | 02°07′ S, 77°44′W | 650 | [6] |
| **Family: Hylidae** |  |  |  |  |  |  |  |
| *Dendropsophus carnifex* | 10 | 5 | RLG | Pichincha | 0°01’S, 78°44’W | 1822-2400 | [5] |
| *Hyloscirtus alytolylax* | 16 | 10 | RLG | Pichincha | 0°01’S, 78°44’W | 1822-2400 | [5] |
| *Hypsiboas boans* | 1 | 0 | PNY | Orellana | 0.6384694°S, | 217 | [7] |
|  |  |  |  |  | -76.1490806°W |  |  |
| *Smilisca phaeota* | 4 | 0 | Bilsa-Jatun Sacha | Esmeraldas | 01°4′06.02′′S, 77°36′42.14′′ W | 450 | [6] |
| **Family: Hemiphractidae** |  |  |  |  |  |  |  |
| *Gastrotheca plumbea* | 4 | 2 | Santo Domingo | Pichincha | 00°05′31.11′′S, 78°29′44.41′′W | 2696 | [6] |
| *Gastrotheca pseustes* | 12 | 12 | La Virgen | Napo | ND | ND | [2] |
|  | 1 | 0 | PCVT | Napo | ND | ND | [2] |
| *Gastrotheca riobambae* | 13 | 7 | Cuendina | Pichincha | 78,4725°O, 0,415118°S | ND | [2] |
|  | 4 | 1 | Quito | Pichincha | 00°1′47.79′′S, 78°27′51.39′′W | 2941 | [6] |
|  | 4 | 1 | San Rafael | Pichincha | 00°17′47.76′′S, 78°27′36.67′′W | 2460 | [6] |
|  | 4 | 2 | Ibarra | Imbabura | 00°17′0′′ N, 78°7′0′′ W | 2192 | [6] |
| *Gastrotheca sp2* | 4 | 1 | Cuenca | Azuay | 02°55′39.94′′S, 78°59′17.32′′W | 2630 | [6] |
|  | 4 | 1 | San Fernando | Azuay | 03°8′52′′S, 79°15′6′′ W | 2665 | [6] |
| *Gastrotheca sp4* | 4 | 1 | Chilla | Oro | 03°27′S, 79°35′24′′ W | 3200 | [6] |
| **Family: Craugastoridae** |  |  |  |  |  |  |  |
| *Pristimantis achatinus* | 7 | 4 | RLG | Pichincha | 0°01’S, 78°44’W | 1822-2400 | [5] |
|  | 4 | 0 | Bilsa-Jatun Sacha | Esmeraldas | 01°4′06.02′′S, 77°36′42.14′′ W | 450 | [6] |
| *Pristimantis appendiculatus* | 23 | 10 | RLG | Pichincha | 0°01’S, 78°44’W | 1822-2400 | [5] |
| *Pristimantis aureolineatus* | 7 | 4 | PNY | Orellana | 0.6384694°S, | 217 | [7] |
|  |  |  |  |  | -76.1490806°W |  |  |
| *Pristimantis calcarulatus* | 30 | 3 | RLG | Pichincha | 0°01’S, 78°44’W | 1822-2400 | [5] |
| *Pristimantis curtipes* | 9 | 1 | DPHT | Chimborazo | 78,72319°O, 2,02687°S | ND | [2] |
|  | 2 | 0 | PCVT | Napo | ND | ND | [2] |
|  | 9 | 0 | La Virgen | ND | ND | ND | [2] |
| *Pristimantis eremitus* | 8 | 6 | RLG | Pichincha | 0°01’S, 78°44’W | 1822-2400 | [5] |
| *Pristimantis eugeniae* | 20 | 13 | RLG | Pichincha | 0°01’S, 78°44’W | 1822-2400 | [5] |
| *Pristimantis festae* | 5 | 3 | PCVT | Napo | ND | ND | [2] |
|  | 21 | 4 | La Virgen | ND | ND | ND | [2] |
| *Pristimantis hectus* | 22 | 6 | RLG | Pichincha | 0°01’S, 78°44’W | 1822-2400 | [5] |
| *Pristimantis illotus* | 1 | 0 | RLG | Pichincha | 0°01’S, 78°44’W | 1822-2400 | [5] |
| *Pristimantis lanthanites* | 1 | 1 | PNY | Orellana | 0.6384694°S, | 217 | [7] |
|  |  |  |  |  | -76.1490806°W |  |  |
| *Pristimantis ockendeni* | 6 | 1 | PNY | Orellana | 0.6384694°S, | 217 | [7] |
|  |  |  |  |  | -76.1490806°W |  |  |
| *Pristimantis parvillus* | 9 | 4 | RLG | Pichincha | 0°01’S, 78°44’W | 1822-2400 | [5] |
| *Pristimantis sobetes* | 17 | 6 | RLG | Pichincha | 0°01’S, 78°44’W | 1822-2400 | [5] |
| *Pristimantis pteridophilus* | 3 | 0 | RLG | Pichincha | 0°01’S, 78°44’W | 1822-2400 | [5] |
| *Pristimantis thymelensis* | 1 | 1 | PCVT | Napo | ND | ND | [2] |
|  | 3 | 0 | La Virgen | ND | ND | ND | [2] |
| *Pristimantis unistrigatus* | 4 | 2 | Quito | Pichincha | 00°1′47.79′′S, 78°27′51.39′′W | 2941 | [6] |
| *Pristimantis waoranii* | 9 | 3 | PNY | Orellana | 0.6384694°S, | 217 | [7] |
|  |  |  |  |  | -76.1490806°W |  |  |
| *Pristimantis w-nigrum* | 21 | 13 | RLG | Pichincha | 0°01’S, 78°44’W | 1822-2400 | [5] |
| **Family: Leptodactylidae** |  |  |  |  |  |  |  |
| *Engystomops petersi* | 6 | 1 | PNY | Orellana | 0.6384694°S, | 217 | [7] |
|  |  |  |  |  | -76.1490806°W |  |  |
| *Leptodactylus discodactylus* | 5 | 2 | PNY | Orellana | 0.6384694°S, | 217 | [7] |
|  |  |  |  |  | -76.1490806°W |  |  |
| *Leptodactylus pentadactylus* | 5 | 4 | PNY | Orellana | 0.6384694°S, | 217 | [7] |
|  |  |  |  |  | -76.1490806°W |  |  |
| *Leptodactylus rhodomystax* | 1 | 1 | PNY | Orellana | 0.6384694°S, | 217 | [7] |
|  |  |  |  |  | -76.1490806°W |  |  |

CCA-Amaru: Centro de Conservación de Anfibios-Amaru.

PNY: Parque Nacional Yasuní.

RLG: Reserva Las Gralarias.

PCVT: Papallacta, Comunidad Valle del Tambo.

DPHT: Desierto de Palmira, hacienda Tabrilla.

References:

1. Ron PSR, Merino A. Declinación de anfibios del Ecuador: informacion general y primer reporte de chytridiomycosis para Sudamerica. 2000;1998: 2–3.

2. Sáenz V. Genética de poblaciones y prevalencia del hongo patógeno Batrachochytrium dendrobatidis en anfibios de Papallacta y Guamaní, Ecuador. 2011.

3. Torres NG. Implementación y evaluación de un ensayo de PCR para la detección del agente causal de la quitridiomicosis. 2019.

4. Merino-Viteri A, Salazar-Valenzuela D. A midaltitude report of Batrachochytrium dendrobatidis in Ecuador. 2007.

5. Guayasamin JM, Mendoza AM, Longo A V., Zamudio KR, Bonaccorso E. High prevalence of Batrachochytrium dendrobatidis in an Andean frog community ( Reserva Las Gralarias , Ecuador ). 2014.

6. Bresciano JC, Salvador CA, Paz-y-Miño C, Parody-Merino AM, Bosch J, Woodhams DC. Variation in the Presence of Anti-Batrachochytrium dendrobatidis Bacteria of Amphibians Across Life Stages and Elevations in Ecuador. Ecohealth. 2015;12: 310–319. doi:10.1007/s10393-015-1010-y

7. McCracken S, P. Gaertner J, Forstner M, Hahn D. Detection of Batrachochytrium dendrobatidis in amphibians from the forest floor to the upper canopy of an Ecuadorian Amazon lowland rainforest. Herpetological Review. 2009.
