# Supplementary material for: Field-based molecular detection of Batrachochytrium dendrobatidis in critically endangered Atelopus toads and aquatic habitats in Ecuador
Source: PLoS One. 2024 Mar 14;19(3):e0299246. doi: 10.1371/journal.pone.0299246 (PMC10939218; doi:10.1371/journal.pone.0299246)
Supplement: Attachment — Submitted filename: renamed_3cd20-REV.docx [file pone.0299246.s005.docx]

Portable field-laboratory for in-situ molecular detection of the lethal fungus ***Batrachochytrium dendrobatidis*** on three critically endangered ***Atelopus*** toad species in Ecuador.

Lenin R. Riascos-Flores^1,2,10^, Julio Bonilla^2^, Leopoldo Naranjo-Briceño^3^, Katherine Apunte-Ramos^4^, Grace C. Reyes-Ortega^5^, Marcela Cabrera^6,7^, José F. Cáceres-Andrade^8^, Andrea Carrera-Gonzalez^4^, Jomira K. Yánez-Galarza^3,5^, Fausto Siavichay Pesántez^9^, Peter Goethals^1^, Jorge Celi^10^, Christine Van der heyden^11^, & H. Mauricio Ortega-Andrade^5,12^*

^1^ Department of Animal Sciences and Aquatic Ecology, Faculty of Bioscience Engineering, Gent University, Coupure Links 653, 9000 Gent, Belgium

^2^ Escuela Superior Politécnica del Litoral, ESPOL/Facultad de Ciencias de la Vida, Centro de Investigaciones Biotecnológicas del Ecuador, Campus Gustavo Galindo Guayaquil, km 30.5, Vía Perimetral – 09–01-5863 – Guayaquil – Ecuador.

^3^Applied Microbiology Research Group, Life Sciences Faculty, Universidad Regional Amazónica IKIAM, Tena, Ecuador.

^4^ Molecular Biology and Biochemistry Lab. Universidad Regional Amazónica IKIAM, Tena, Ecuador

^5^ Biogeography and Spatial Ecology Research Group, Life Sciences Faculty, Universidad Regional Amazónica IKIAM, Tena, Ecuador

^6^ Laboratorio Nacional de Referencia del Agua, Universidad Regional Amazónica Ikiam, Tena 150150, Ecuador.

^7^ University of Valencia, 46980 Paterna, Spain.

^8^ Parque Nacional Cajas ETAPA EP, Cuenca, Ecuador.

^9^ Centro de Conservación de Anfibios AMARU, Cuenca, Ecuador.

^10^ Water and Aquatic Resources Research Group, Earth and Water Sciences Faculty, Universidad Regional Amazónica Ikiam, Tena, Ecuador.

^11^ HOGENT - Health and Water Technology Research Centre, Valentin Vaerwyckweg 1, 9000 Gent, Belgium.

^12^ Herpetology Division, Instituto Nacional de Biodiversidad, Quito, calle Rumipamba 341 y Av. de los Shyris, Pichincha, Ecuador.

* Corresponding author

E-mail: mauricio.ortega@ikiam.edu.ec

Author contribution

Conceptualization, methodology, formal analysis, investigation, LR, HMOA, JB, LNB, KAR, GCRO, JY, ACG and CVdH; validation, formal analysis, data curation, visualization, supervision, LR, HMOA, JB, LNB, GCRO, ACG and CVdH; writing original draft preparation, writing—review and editing, LR, HMOA, JB, LNB, KAR, GCRO, MC, JFCA, ACG, JY, FSP, PG, JC and CVdH; project administration, funding acquisition, HMOA, JC, CVdH. All authors have read and agreed to the published version of the manuscript, and declared not conflict of interest.

Abstract

*Batrachochytrium dendrobatidis* (Bd) is a lethal fungal species that parasites vertebrates and is associated with declining populations of endemic amphibian species worldwide. Management strategies would greatly benefit from sensitive and fast detection methods that can be applied broadly in field, which include DNA methods. In this study, we tested the reliability of a portable field-laboratory (PL) to detect the presence of Bd in native populations of three critically endangered species of *Atelopus* toads in Ecuador. We collected swabs from individuals of the endangered toads *Atelopus balios, A. nanay,* and *A. bomolochos,* and water samples (environmental DNA) from different streams in the Andes and coastal Ecuadorian areas. Samples were processed for on-field molecular analyses, and results were obtained within eight hours after sampling. Prevalence of Bd was detected in eDNA and swabs in the monitored ecosystems (5/11 water samples and 10/12 amphibians swab samples). eDNA results obtained with the portable field laboratory were 100% like those obtained under on-campus laboratory conditions. Our results highlight the potential of a PL using DNA-based monitoring to assess the presence and prevalence of Bd in amphibians and aquatic ecosystems, which would strengthen the National Action Plan for the Conservation of Ecuadorian Amphibians and global efforts to deal with this lethal fungus and invasive species in general. Furthermore, our field lab approach quickly leads to reliable and low-cost monitoring of tropical invasive and zoonotic species in remote areas, in the context of a pandemic.

Keywords

# Environmental DNA, metabarcoding, molecular ecology, Invasive species, pathogens

# Introduction

Amphibians (frogs, toads, salamanders, and caecilians) most infectious and mortal registered disease is the chytridiomycosis produced by the zoonotic fungus *Batrachochytrium dendrobatidis* (Bd) and *Batrachochytrium salamandrivorans* (Bsal). After the massive mortality event of frogs in Australia and Central America [1] and the reports of massive propagation worldwide, Bd has been reported in over 700 species of amphibians [2]. Bd is considered responsible for the decline of about 501 amphibian species and 90 possible extinctions worldwide [3]. Most of the mass mortality and extinction of amphibians by Bd has occurred found in the American and Oceania continents [4]. The largest amphibian die-off worldwide occurred in the 1980s, while in South America, the second-biggest mass die-off event occurred in the mid-2000s [3, 5].

Bd acts like an epidemic disease, and is spread around ecosystems mainly by human activities and vector species (e.g. fish, arthropods and crayfish) in a wide range of hosts [6, 7]. The most known way of distribution of Bd is through water sources [8]. Bd is transmitted via animal contact or waterborne motile zoospores [9]. Bd is detected in larvae, metamorphs, and adult amphibian hosts using various techniques such as visual inspection of tadpoles, fungal culture, light microscopy, immune histochemistry, electron microscopy, immuno-electron microscopy and polymerase chain reaction (PCR). However, with the advances in genetics and genomic technologies, molecular techniques (PCR and qPCR) have become the most common and reliable assay method for identifying Bd [10, 11].

*Atelopus* toads (Anura: Bufonidae) are one of the most threatened groups worldwide, with at least 80% of their species considered in a category of extinction [12]. In Ecuador, this percentage increases to 100% of the 25 species reported species [13]. Three of the critically endangered *Atelopus* toads in the country are *A. nanay* and *A. bomolochos,* endemic of the Central Andes highlands in Ecuador, and *A. balios,* endemic of the coastal region. Their habitat has been severely damaged by synergetic effects caused by habitat loss, climate change and emergent diseases [14, 13, 15]. The first case of chytridiomycosis in South America was discovered on a specimen of *A. bomolochos* in 1980 [16]. Furthermore, *A. bomolochos* was considered extinct for 28 years until the re-discovery of a few individuals in 2015. *Atelopus nanay* lives only in the highlands of the Cajas National Park, Azuay, Ecuador [17]. For both species, the distribution of habitat is restricted to about 60 km^2^, which promotes an increase in the risk of extinction [13]. In the last five years, Bd cases have been reported in those very restricted and uncommon populations [18, 19], indicating the persistence of the pathogen.

Worldwide, monitoring the abiotic and biotic state of habitats and species that could be infected with Bd is considered the first step towards understanding the mechanism to control and manage the diseases [6]. In contrast, the limited monitoring initiatives in developing countries does not match the with high levels of regional biodiversity and the number of species considered as threatened by Bd [12, 20]. Other challenges in developing countries are the higher cost of equipment, the limited sealing of reagents and the complex bureaucratic import processes of the country. In this context, biomonitoring based on portable and low cost sequencing devices facilitate assessing biological biodiversity in hot-spots around the world [21, 22].

This study aims to test the reliability and accuracy of a portable field-laboratory to detect the presence of Bd in native populations of three threatened Atelopus toad species, in the Central Andean highlands and coastal Ecuador. The results are framed in a context of the local environmental field conditions.

# Materials and methods

## ***Study area***

Sampling sites were selected based on to the previous reports on the distributional range of *Atelopus* species [13, 17]. *A. balios* was previously reported in Western Piedmont Forest in Coastal Ecuador, while *A. nanay* and *A. bomolochos* were reported in natural Paramo areas in the Cajas National Park and nearby areas in the highlands (Figure 1). SWABs and water samples were collected between 4-8th October 2020 in two localities from Azuay province: Cerro Negro, 3.15675° S, 78.84538° W, 3064 m.a.s.l. (Site A), Sig Sig - Angas, Parque Nacional Cajas, 2.88337° S, 79.30685° W, 3800 m.a.s.l. (Site B); and two localities from Guayas province: Cerro Las Hayas, 2.72452° S, 79.61892° W, 107 m a.s.l. (Site C), Cerro San Miguel, Estero Arenas, 2.75077° S, 79.01269° W, 203 m.a.s.l. (Site D; Supplementary Table S1). A threat model to assess the Environmental Risk Surface (ERS) was based on the Red List of Ecuadorian Amphibians dataset for the study area [13]. Coastal Ecuador, while *A. nanay* and *A. bomolochos* were reported in natural Paramo areas in the Cajas National Park and nearby areas in the highlands (Figure 1). SWABs and water samples were collected between 4-8th October 2020 in two localities from Azuay province: Cerro Negro, 3.15675° S, 78.84538° W, 3064 m.a.s.l. (Site A), Sig Sig - Angas, Parque Nacional Cajas, 2.88337° S, 79.30685° W, 3800 m.a.s.l. (Site B); and two localities from Guayas province: Cerro Las Hayas, 2.72452° S, 79.61892° W, 107 m a.s.l. (Site C), Cerro San Miguel, Estero Arenas, 2.75077° S, 79.01269° W, 203 m.a.s.l. (Site D; Supplementary Table S1). A threat model to assess the Environmental Risk Surface (ERS) was based on the Red List of Ecuadorian Amphibians dataset for the study area [13]. The Ministry of Environment of Ecuador for providing the research permits “MAE-DNB-CM-2017-0062" and “MAATE-DBI-CM-2021-0177”.

***Amphibian sampling***

No specimens were euthanized. Handling specimens were directly made with new nitrile gloves in the field, and skin sampling was developed with a sterile CITOSWAB SERIES® microbiological collection and transport system (Amies Charcoal Gel 2120-0025). The individuals were sprayed with sterilized Milli Q water, and then their skin –belly, groin, legs, feet, and hands– was swabbed with one swab five times. Finally, swabs were stored in a 1.5mL microtube with 400µL of lysis buffer (Tris-HCl 0.18 M; EDTA 10 mM, SDS 1 %, pH 8.2). After sampling, specimens were released on the same place of capture.

*Water collection and physical-chemical analysis*

Before the eDNA sampling, all materials were disinfected in the laboratory using a sodium hypochlorite solution (1/5 of the commercially available solution) adapted from *Geerts* et al. [23]. A blank sample (bottle with distilled and sterilized water) was processed for each site to assess potential contamination. Sterilization of materials in the field, such as scissors and tweezers, was done through rinsing utensils with ethanol 96% and subsequent sterilized with fire.

One litre of water was taken for each location at three sites (about 350 ml per site), and 1 L was filtered over a 0.45 μm pore-sized nitrocellulose filter with 47 mm diameter (Porafil®). Filtering was done using a Gast DOA-P704-AA High-Capacity Vacuum Pump and sterilized Nalgene filtration devices (Thermo Fisher Scientific). For blank samples, 1 L of sterilized distilled water was filtered prior to the samples; this process was repeated for each location. Before extracting eDNA in the field-lab, filters were divided into two halves. One half was used for DNA isolation in the field with portable devices, while the other half was stored at -20 °C, and the DNA was isolated at the university laboratory. Each half of the filter was cut into small 1 to 2 mm bands. These bands were handled with sterilized tools and were stored in sterile 1.5 ml Eppendorf tubes. Eppendorf tubes were cooled or frozen for field and university laboratories, respectively. Between filters procedure, tools were sterilized by rinsing with 95% ethanol, followed by burning the residual ethanol.

From each sample site, water samples (500 ml) for physical-chemical analyses were collected in sterilized plastic bottles, frozen and transported to the university laboratory for further processing. From the eighteen physical-chemical parameters were analysed (See Supplementary Table ST2 for details), twelve parameters such as pH, Dissolved Organic Carbon, Turbidity, Chlorides, Nitrites, Nitrates, Phosphates, Sulfates, Sodium, Potassium, Magnesium, Calcium were used to do elaborate a cluster analysis. Principal component analysis was performed in PAST software [24].

*DNA isolation from Swabs*

Disposable gloves were worn and changed between samples during the lab work and sample preparation. At the portable field-laboratory and to prevent contamination, a separate table was dedicated for preparing PCR mix, while gel electrophoresis and PCR were each done on separate space (Figure 2). Additional decontamination practices were used for each isolation [25, 23]. Extraction of fungal metagenomic DNA from swabs was performed using the Wizard® Genomic DNA Purification Kit (Promega Cat. # A1120). Swabs were mixed in the lysis buffer (Tris-HCl 0.18 M; EDTA 10 mM; SDS 1%, pH 8.2) with the use of sterile tweezers. 5µL of Proteinase K (50µg/µL) (Invitrogen Cat. # QS0511) was added and samples were mechanically disrupted by shaking a sterile pestle against the walls of the microtube. After, vortex for 20 s at maximum speed (Vortex V-1 plus), the tubes were incubated in a thermoblock at 65°C for 30 min. Then, 2.5 µL RNase (10 mg/mL) (Thermo Scientific, Cat. # EN0531) was added and mixed by pipetting the solution and incubated at 37°C for 30 min. For cells and protein precipitation, 100µL of Protein Precipitation Solution was added and vortexed for 20s followed by incubation for 10 min on ice. After centrifugation at 12000 rpm for 5 min, the supernatant was recovered and transferred to a clean sterilized 1.5 mL microtube. To precipitate the DNA, 40 µL of Sodium acetate (3M, pH 5.2) and 1000µL of absolute ethanol were added and centrifuged for 5 min at 12000rpm. The supernatant was discarded, and the remaining droplets were removed with a clean, sterile micropipette tip after spinning. The microtube was subsequently dried in a thermoblock at 60°C until no liquid residue was observed. Finally, the DNA was resuspended in 25 µL of sterilized MilliQ H_2_O.

*DNA isolation from water samples*

For the extraction of DNA from the first-half nitrocellulose snippets, we applied an adapted version of the Epicenter Masterpure DNA Purification Kit used by Riascos *et al.* [25] for eDNA extraction of water samples of Andean lakes. The adaptations were: doubling the volume of Tissue and Cell Lysis Solution, proteinase K and RNase, adding two sterile glass beads after homogenizing the samples, and changing the incubation time with proteinase K to thirty minutes with vortex every 10 minutes. The time of incubation with RNAse at 37 °C was decreased to 10 minutes, and 175 μL MPC Protein Precipitation Reagent was added. The obtained DNA pellet was resuspended in 40 μL sterilized demi-water instead of Epicentre’s TE buffer.

*DNA, PCR products and electrophoresis*

In the field laboratory, we assessed the integrity of the DNA by electrophoresis in a 0.8% agarose gel with 1X TBE and Gel Star™ Nucleic Acid Stain diluted 1:10,000 (Lonza, Lonza Rockland) to stain DNA. For the identification of Bd presence, we used the specific primers Chytr ITS1-3 (5'-CCTTGATATAATACAGTGTGC- CATATGTC-3') and Chytr - 5.8S (5'-AGCCAAGAGATCCGTTGTCAA-3') to detect Bd DNA [26]. Reactions contained 7.5µL of sterilized MilliQ H_2_O, 1µL of each primer at a concentration of 10mM, 12.5µL of TaqMan Environmental Master Mix 2.0 (Applied Biosystems, Cat. # 4396838), and 3µL of DNA for a total volume of 25µL per reaction. Thermal cycling was done in a miniPCR thermal cycler programmed with the following conditions: initial denaturation at 95°C for 120s, 35 cycles of denaturation at 95°C for 60s, annealing at 60°C for 30s, extension at 72°C for 30s and final extension at 72°C for 300s. After amplification in the field, electrophoresis of PCR products was performed in a blueGel electrophoresis system using a 2% agarose gel in 1x Tris/Borate/EDTA solution (TBE buffer) with GelStar™ Nucleic Acid Stain diluted 1:10,000 (10,000X in DMSO) (Lonza, Lonza Rockland).

To extract eDNA from the second halves of the filters, a similar DNA extraction and PCR protocol to the one of the from field-lab was used in the university laboratory. PCR products were examined by electrophoresis in a 2% agarose gel with 1X TBE. The first slot was loaded with 3 μL of 100 bp DNA Ladder (Promega) and 2 μL of Blue/Orange 6X Loading Dye (Promega); other slots were filled with a mix of 10 μL DNA PCR product and 2 μL of Blue/Orange 6X Loading Dye (Promega). The loaded gel ran for 25 minutes at an electric potential difference of 135 V. After electrophoresis, the gel was stained with GelStar™ Nucleic Acid Stain diluted 1:10000 (Lonza, Lonza Rockland). If samples showed a 146 bp band, the amplification of Bd DNA was considered successful. As negative controls showed no amplified products, PCR results from environmental samples were considered true positives or negatives compared to the controls. A standardized protocol to collect, isolate and amplify DNA, eDNA, from water samples to detect Bd with portable devices is detailed in Supplementary Material.

*Sequencing products*

To evaluate the reliability and accuracy on BD detection we randomly selected samples for Sanger sequencing and amplified PCR products. Positive amplicons were sent for sequencing to Macrogen facilities (Seoul, Korea) to confirm the cloned sequences of the Bd nuclear ribosomal region. Sequences were manually trimmed and cleaned in Geneious Prime® (ver. 2020.2.2). Amplified PCR products were confirmed positives via sequencing and BLASTn algorithm (Update date: 2022/10/14, See Supplementary Material SM2, Supplementary Figure 1). Accession numbers of sequences are detailed in Supplementary Table 1 (*SUB12413894, provisional).

Results

*Detection of Bd from swabs in endangered amphibians*

During monitoring, twelve individuals of *Atelopus* were detected and swabbed (see Supplementary Table S1 for sampling code). Ten out of twelve (83%) swabs used to collect DNA from the skin of amphibians were positive for Bd (see Table 1), showing a signal at 146 bp as expected by targeted amplicon amplification in PCR. In Cerro Negro, two out of two *A. bomolochos* were positive for Bd. In the Cajas National Park-Angas locality, two of three specimens of *A. nanay* resulted positive for Bd, including a dead specimen found below a rock in the stream (HMOA 2399). From seven specimens of *A. balios* assessed in Cerro de Hayas and Estero Arenas, six tested positive for Bd.

*Presence of Bd in water ecosystems*

Water sample codes and dates of DNA isolation are presented in table S3. Five out of twelve (42%) filtered water samples in the field laboratory showed a positive signal for Bd. The procedure was repeated at the university lab, where all positive and negative field reactions were confirmed (see electrophoresis results in Figure S1). Results were confirmed by PCR amplification and via Sanger sequencing.

Locations with a higher prevalence of Bd occurred in the Cajas National Park around 3,000 m.a.s.l. (Cerro Negro 80% and Angas 66%), where non-human disturbance was observed in these places. Sites with less amplification of Bd in water samples (Cerro de Hayas and Estero Arenas, 50% each) were located in the Coastal lowlands at about 300 m asl. For the last two places, the human disturbance was high with crops and cattle ranching, close to the monitoring sites.

Principal component analysis (PCA) showed a total contribution of 97.9% with the two principal components (PC) (see Figure 3, Raw data in Supplementary Material SM3). Sulfates, calcium and sodium were the main parameters associated positively with PC1, whereas calcium and sodium were negatively associated with PC2. Turbidity and chloride were positively related to PC1 and PC2. All other parameters resulted in low values of contribution in the PCA. Highland locations (Cerro Negro and Sig Sig) were mostly associated with calcium and sodium. In contrast, lowland localities (Cerro de Hayas and Estero Arenas) were associated with sulfate, turbidity and chloride (Figure 3).

Discussion

*The endangered toads Atelopus and the portable laboratory as an instrument for research in conservation*

Herein we report the results of the use of a field laboratory to detect Bd in four sites in the Ecuadorian Andes and Coastal lowlands. Our results demonstrate that a basic-equipped portable laboratory (i.e., PCR instead of qPCR or sequencing) in combination with our sample processing methods are accurate and rapid for detecting Bd in remote areas of three critically endangered species from different environments with varying levels of disturbance (Figure 1, Table 1).

*Atelopus* frogs assessed herein correspond to critically endangered toads [13]. Before the recent rediscovery of *A. balios* and *A. bomolochos*, both species were registered for the last time in 1995 and 1990, respectively [17, 16]. The locations sampled for *A. balios* and *A. nanay* are the last known habitats in which these species were reported. For the former species, besides the infection of Bd, several other threats have been identified. For example, in Cerro de Hayas and Estero Arenas, habitat loss (deforestation), cattle grazing, and chemicals used for agriculture were observed during the monitoring.

Herein, we found positive Bd results for each *Atelopus* species swabbed using portable devices. This finding suggests that field-based equipment are potential tools to fast pathogen detection on endangered amphibians, which is crucial to implement pest management and control strategies.

Before this study, the main limiting factors to designing and deploying the study of Bd amphibian pandemic in the country were: the absence of field labs, lack of expertise, and standardized field protocols for molecular analysis. For instance, in Ecuador, from the 635 native amphibian species registered [13] thirty-six species are reported with Bd (Supplementary Table ST3)*.* These reports belong to seven studies in 5 families and 44 evaluated species in 10 of the 24 provinces of Ecuador. The next step towards the national conservation goals highlights the need for the implementation of portable laboratories and molecular tools for real-time sequencing (i.e., MinION Oxford Nanopore Technology), to enable efficient and fast detection of emergent agents like Bd, e.g., Ranavirus.

*Differences in PCR amplification for water samples.*

Although the water sample results were equal for the field-portable and university laboratories, negative results (no amplification) were observed in different locations. These negatives could be explained by different factors such as the absence of the target Bd at the monitoring site, sample concentration, amplification methodologies used, PCR or the influence of abiotic and biotic factors [27-29] On the other hand, differences in the disturbance range between locations could also affect the results. For example, in Coastal sites, traditional crops (cacao and banana), cattle and other human pressures (Figure 1) were observed. The number of negatives in the eDNA water samples were higher in comparison to the Andean sites, which were in areas with low human impact (Figures 1,3).

Previous studies demonstrate that chytrid pathogens and amphibian hosts can be affected by factors such as pesticides, which may cause zoospore mortality or reduced zoospore production and increased host susceptibility to infection and disease [30, 31]. Moreover, previous studies in Ecuador demonstrate the presence of forbidden pesticides (POPs) in aquatic ecosystems and remote areas [32-34] that can negatively influence eDNA stability. As an example, organophosphates used to kill pests in crops could promote the increase of reactive oxygen species (ROS) in water and thus contribute to eDNA damage [35]. Besides, presence of pesticides could exert an inhibitory effect on PCR performance. In that sense, certain compounds such as dithiocarbamates completely inhibit amplification [36]. Furthermore, highland localities were associated with calcium and sodium, whereas lowlands were associated with sulfates, chloride and turbidity (Figure 3). In addition, the highlands presented a higher concentration of DOC (2.84 and 3.89 mg/L). Eichmiller *et al. [37]* reported that DNA binds readily and strongly to humic substances, which are part of DOC in aquatic environments, and protects DNA from degradation. According to the results obtained in this study, the physiochemical parameters of the water could impact the recovery percentage of DNA. In this context, more studies are necessary to understand the presence, degradation and distribution of eDNA in natural or affected tropical ecosystems, as well as its reliability in detecting and identifying the presence of invasive species directly in field conditions in tropical ecosystems.

*Needs, challenges and advantages of portable molecular laboratories in remote areas*

The capacity to early detect invasive or pathogenic species in the field with molecular accuracy is a key aspect of monitoring and surveillance for biodiversity conservation. However, molecular techniques commonly involved in pathogen detection, such as DNA extraction and PCR assays, commonly require laboratories with complex and expensive equipment. In this sense, field-based and portable devices have been reported as promising tools for the rapid and precise detection of emerging pathogens [38]. The advantages of using portable equipment are easy transportation to the sampling site, convenient handling, and immediate results [39].

On-site filtering of the water samples and on-site eDNA isolation close to the amphibian collection sites reduced the time between sampling and filtering considerably, thus increasing the possibilities to maximize eDNA recovery from fresh samples [40]. Furthermore, using portable equipment allows to mitigate the influence of certain harmful factors on the viability of eDNA samples. Specifically, the maintenance of the cold chain during eDNA sample transportation from remote localities to the laboratory is considered a challenge because of temperature impacting the decrease of DNA concentration [37]. For instance, for this specific study in Ecuador, storing and transporting water samples for environmental DNA analysis to a research facility or University laboratory took two to three days by road. Other advantages are the use of basic standardized short-term molecular techniques in low-cost equipment for DNA isolation and conventional PCR, which can overcome many of the problems related to the scarcity of high-cost laboratory equipment in countries with emerging economies, which helps increase awareness and access to modern-portable technology, further building and strengthening research capacities in developing countries.

In Ecuador, like in other tropical countries characterized by developing economies, equipment and consumables from local suppliers can cost two to five times more than the original price in the USA or Europe [41]. Moreover, limited access to grant opportunities, inadequate budgets, and lack of laboratory infrastructure and equipment contribute to the dearth of monitoring to evaluate the impact of invasive species in Ecuador. For example, a typical grant is about 5,000 to 40,000 US dollars for the total cost of project, and the local prices of equipment for a basic molecular laboratory can range from 10,000 to 30,000 US dollars. With these considerations, about 80 – 90 % of the project budget would have to be allocated to equipment purchase, limiting budget allocation for sampling, reagents, and in some cases, external services (i.e., Sanger sequencing), thus making the project unfeasible. The portable equipment used in our study, the miniPCR DNA Discovery System (approx. 2,000 US dollars), has a low purchase cost compared to the standard university laboratory equipment. In addition, access to the available product portfolio (reagents, equipment, laboratory materials) overseas is limited in Ecuador due to high import taxes, few distribution companies, and few available brands.

Portability, local availability and affordability were the main reasons for selecting the equipment used during this research. Implementing low-cost technologies can facilitate sampling campaigns and become the fundamental tool to delimit risk assessment and risk management, preserve endangered species (e.g., treatment of infected individuals), and elaborate reintroduction or eradication (invasive species) plans [42, 43]. Therefore, it is crucial to apply methods to integrate the use of low-cost technologies in the study of biological invasions, invasive species, emerging infectious diseases, and zoonosis [13] in a context of a fast and effective management of lethal fungi and biological invasions that are global threat to diversity [44].

Conclusions

Worldwide, the impacts of Bd and chytridiomycosis in frog communities remain mostly unstudied. Although different strategies may be implemented to control Bd prevalence among analysed amphibians, the first step requires a detailed identification of the areas and infected species. Due to the high costs of equipment and consumables for studying the presence or absence of Bd in frog species in Ecuadorian ecosystems, we have assessed the capacity of implementing a low-cost portable laboratory that combined with eDNA water samples, can yield a fast, sensitive, and highly reliable approach for the detection of Bd when compared to the traditional on-campus laboratory facilities. Our main conclusions are: (i) Results to detect Bd with portable devices are comparable to techniques applied in a university laboratory, but with faster processing times for samples in the field and at a lower cost by using the portable laboratory equipment; (ii) Populations assessed showed 83% of Bd prevalence in specimens analysed, and 42% of water eDNA samples were positive for Bd; and (iii) The use of technologies that provide information based on fast laboratory protocols and low-cost equipment are now accessible for researchers to fill the gap in Bd detection. Improving these protocols with cutting-edge biotechnology for real-time sequencing, we contribute to establishing a method for portable biodiversity monitoring of emergent diseases and threatened species as an integral part of future biodiversity genomics applied to conservation.

Acknowledgments

We gratefully acknowledge the “Networks 2019 phase 2 Ecuador biodiversity project”, the VLIR-UOS South Initiative ZEIN 2014Z15 “DNA-based monitoring for assessing the effect of invasive species on aquatic communities in the Amazon basin of Ecuador”, the VLIR-UOS South Initiative EC 2020 SIN 277B125 “Biomonitoring of aquatic environments in the Amazon using environmental DNA”, the “Conservation of Ecuadorian Amphibians and access to genetic resources-PARG” managed by the Ministry of Environment and Water of Ecuador, the project “On the quest of the golden fleece in Amazonia: The first herpetological DNA - barcoding expedition to unexplored areas on the Napo watershed, Ecuador”, the World Academy of Sciences. We also thank Luis Oyagata, Jackeline Arpi, Naty Aguilar, Evelyn Ocampos, Alex Arias, Walter Quilumbaquin and Noemí Torres for their support and assistance in field and laboratory work.

References

[1.	Berger L, Speare R, Daszak P, Green DE, Cunningham AA, Goggin L, et al. Chytridiomycosis Causes Amphibian Mortality Associated with Population Declines in the Rain Forests of Australia and Central America. Pnas. 1998;95(15):9031-6.](https://www.iucnredlist.org)

.

13.	Ortega-Andrade HM, Rodes Blanco M, Cisneros-Heredia DF, Guerra Arévalo N, López de Vargas-Machuca KG, Sánchez-Nivicela JC, et al. Red List assessment of amphibian species of Ecuador: A multidimensional approach for their conservation. PLOS ONE. 2021;16(5):e0251027. doi: 10.1371/journal.pone.0251027.

14.	Menéndez-Guerrero PA, Davies TJ, Green DM. Extinctions of Threatened Frogs may Impact Ecosystems in a Global Hotspot of Anuran Diversity. Herpetologica. 2020;76(2):121-31. doi: 10.1655/0018-0831-76.2.121.

15.	Menéndez-Guerrero PA, Graham CH. Evaluating multiple causes of amphibian declines of Ecuador using geographical quantitative analyses. Ecography. 2013;36(7):756-69. doi: 10.1111/j.1600-0587.2012.07877.x.

16.	Ron SR, Merino A. Amphibian declines in Ecuador: overview and first report of chytridiomycosis from South America. Froglog. 2000;42(2000):2-3.

17.	Ron SR, Merino-Viteri A, Ortiz DA. Anfibios del Ecuador. Version 2023.0. Quito, Ecuador: Museo de Zoología, Pontificia Universidad Católica del Ecuador; 2023 [15/04/2023]. Available from: <https://bioweb.bio/faunaweb/amphibiaweb>.

18.	Cáceres-Andrade J. Análisis Exploratorio de datos para desarrollar propuestas de conservación de la comunidad de Anfibios referentes al Batrachochytrium dendrobatidis en el Parque Nacional Cajas, Cuenca-Ecuador.: Universidad del Azuay; 2014.

19.	Torres-Sarango N. Implementación y evaluación de un ensayo de PCR para la detección del agente causal de la quitridiomicosis: Universidad de Cuenca; 2019.

20.	Deknock A, Goethals P, Croubels S, Lens L, Martel A, Pasmans F. Towards a food web based control strategy to mitigate an amphibian panzootic in agricultural landscapes. Global Ecology and Conservation. 2020;24(2020):e01314-e. doi: 10.1016/j.gecco.2020.e01314.

21.	Krehenwinkel H, Pomerantz A, Prost S. Genetic Biomonitoring and Biodiversity Assessment Using Portable Sequencing Technologies: Current Uses and Future Directions. Genes. 2019;10(11):858. PubMed PMID: doi:10.3390/genes10110858.

22.	Fujiyoshi S, Yarimizu K, Miyashita Y, Rilling J, Acuña JJ, Ueki S, et al. Suitcase Lab: new, portable, and deployable equipment for rapid detection of specific harmful algae in Chilean coastal waters. Environmental Science and Pollution Research. 2021;28(11):14144-55. doi: 10.1007/s11356-020-11567-5.

23.	Geerts AN, Boets P, Van den Heede S, Goethals P, Van der heyden C. A search for standardized protocols to detect alien invasive crayfish based on environmental DNA (eDNA): A lab and field evaluation. Ecological Indicators. 2018;84(2017):564-72. doi: 10.1016/j.ecolind.2017.08.068.

24.	Hammer Ø, David A, Harper, Ryan PD. Past: Paleontological Statistics Software Package for Education and Data Analysis. Palaeontologia Electronica. 2001;4(1):178-.

25.	Riascos-Flores L, Geerts A, Oña T, Goethals P, Cevallos-Cevallos J, Berghe WV, et al. DNA-based monitoring of the alien invasive North American crayfish Procambarus clarkii in Andean lakes (Ecuador). Limnologica. 2018;70(2018):20-5.

26.	Shin J, Bataille A, Kosch TA, Waldman B. Swabbing often fails to detect amphibian chytridiomycosis under conditions of low infection load. PLoS One. 2014;9(10): e111091. doi: 10.1371/journal.pone.0111091.

27.	Taberlet P, Bonin A, Zinger L, Coissac E. Environmental DNA: For biodiversity research and monitoring: Oxford University Press; 2018. 247 p.

28.	Spens J, Evans AR, Halfmaerten D, Knudsen SW, Sengupta ME, Mak SST, et al. Comparison of capture and storage methods for aqueous macrobial eDNA using an optimized extraction protocol: advantage of enclosed filter. Methods in Ecology and Evolution. 2017;8(5):635-45. doi: 10.1111/2041-210X.12683.

29.	Stewart KA. Understanding the effects of biotic and abiotic factors on sources of aquatic environmental DNA. Biodiversity and Conservation. 2019;28(5):983-1001. doi: 10.1007/s10531-019-01709-8.

30.	Hanlon SM, Kerby JL, Parris MJ. Unlikely remedy: Fungicide clears infection from pathogenic fungus in larval southern leopard frogs (lithobates sphenocephalus). PLoS ONE. 2012;7(8):1-8. doi: 10.1371/journal.pone.0043573.

31.	McMahon TA, Brannelly LA, Chatfield MWH, Johnson PTJ, Joseph MB, McKenzie VJ, et al. Chytrid fungus Batrachochytrium dendrobatidis has nonamphibian hosts and releases chemicals that cause pathology in the absence of infection. Proceedings of the National Academy of Sciences of the United States of America. 2013;110(1):210-5. doi: 10.1073/pnas.1200592110.

32.	Andrée DC, Marie Anne Eurie F, Niels DT, Isabel GA, Arne D, Wout VE, et al. From field to plate: Agricultural pesticide presence in the guayas estuary (Ecuador) and commercial mangrove crabs. Environmental Pollution. 2021;289(2021):117955. doi: 10.1016/j.envpol.2021.117955.

33.	Deknock A, De Troyer N, Houbraken M, Dominguez-Granda L, Nolivos I, Van Echelpoel W, et al. Distribution of agricultural pesticides in the freshwater environment of the Guayas river basin (Ecuador). Science of the Total Environment. 2019;646(2019):996-1008. doi: 10.1016/j.scitotenv.2018.07.185.

34.	Riascos-Flores L, Bruneel S, Van der Heyden C, Deknock A, Van Echelpoel W, Forio MAE, et al. Polluted paradise: Occurrence of pesticide residues within the urban coastal zones of Santa Cruz and Isabela (Galapagos, Ecuador). Science of the Total Environment. 2021;763(2021):142956. doi: 10.1016/j.scitotenv.2020.142956.

35.	Pourmoghadam MN, Poorbagher H, de Oliveira Fernandes JM, Jafari O. Diazinon negatively affects the integrity of environmental DNA stability: a case study with common carp (Cyprinus carpio). Environmental Monitoring and Assessment. 2019;191(11). doi: 10.1007/s10661-019-7816-2.

36.	Farag NS, Balabel NMA. Effect of pesticide(s) carryover on results of polymerase chain reaction and multiplex-PCR for diagnosis of potato bacterial pathogens. Asian Journal of Plant Pathology. 2014;8(1). doi: 10.3923/ajppaj.2014.1.9.

37.	Eichmiller JJ, Best SE, Sorensen PW. Effects of Temperature and Trophic State on Degradation of Environmental DNA in Lake Water. Environmental Science and Technology. 2016;50(4). doi: 10.1021/acs.est.5b05672.

38.	González-González E, Mendoza-Ramos JL, Pedroza SC, Cuellar-Monterrubio AA, Márquez-Ipiña AR, Lira-Serhan D, et al. Validation of use of the miniPCR thermocycler for Ebola and Zika virus detection. PLoS ONE. 2019;14(5). doi: 10.1371/journal.pone.0215642.

39.	Guevara EE, Frankel DC, Ranaivonasy J, Richard AF, Ratsirarson J, Lawler RR, et al. A simple, economical protocol for DNA extraction and amplification where there is no lab. Conservation Genetics Resources. 2018;10(1). doi: 10.1007/s12686-017-0758-5.

40.	Majaneva M, Diserud OH, Eagle SHC, Boström E, Hajibabaei M, Ekrem T. Environmental DNA filtration techniques affect recovered biodiversity. Scientific Reports. 2018;8(1):1-11. doi: 10.1038/s41598-018-23052-8.

41.	Ciocca DR, Delgado G. The reality of scientific research in Latin America; an insider’s perspective. Cell Stress and Chaperones. 2017;22(6):847-52. doi: 10.1007/s12192-017-0815-8.

42.	Ogden NH, Wilson JR, Richardson DM, Hui C, Davies SJ, Kumschick S, et al. Emerging infectious diseases and biological invasions: a call for a One Health collaboration in science and management. Royal Society Open Science. 2019;6(3):181577.

43.	DiRenzo GV, Chen R, Ibsen K, Toothman M, Miller AJ, Gershman A, et al. Investigating the potential use of an ionic liquid (1-Butyl-1-methylpyrrolidinium bis(trifluoromethylsulfonyl)imide) as an antifungal treatment against the amphibian chytrid fungus, Batrachochytrium dendrobatidis. PLoS ONE. 2020;15(4):1-18. doi: 10.1371/journal.pone.0231811.

44.	Falaschi M, Melotto A, Manenti R, Ficetola GF. Invasive Species and Amphibian Conservation. Herpetologica. 2020;76(2):216-27, 12.

Figures

Figure 1. Sampling location in Central Andes of Ecuador. Upper maps: red dots represent the sampling points in each study area. Lower map: Threat model [13] of the study area with the Environmental Risk Surface (ERS) index, location of sampling points (white dots) and the Cajas National Park (white diagonal lines) in Central Andes of Ecuador (red rectangle). ERS= Red color represents a high threat index, whereas blue color represents a low threat index.

Figure 2. A detail of the portable Lab. An example of the distribution of working areas of the portable field-laboratory mounted in a hostel in Cerro de Hayas, Guayas province, Ecuador

Figure 3. Physical-Chemical water analysis. Contribution of physical-chemical parameters to the environmental variation and clustering of sampled localities resulted by Principal Component Analysis.

Tables

Table 1. Field results of water samples and amphibian swabs from four locations in Central Andes of Ecuador.

|  |  |  | SWAB samples | | |  | Water samples | | |
| --- | --- | --- | --- | --- | --- | --- | --- | --- | --- |
| Code | Location Name | ERS | Species | # Samples | PCR positive for Bd |  | Disturbance | # Samples | PCR positive for Bd |
| A | Cerro Negro | 0.015 | *A. bomolochos* | 2 | 2 |  | None | 4 | 3 |
| B | Cajas National Park/Sig Sig-Angas | 0.022 | *A. nanay* | 3 | 2 |  | None | 3 | 2 |
| C | Cerro de Hayas | 0.036 | *A. balios* | 5 | 4 |  | Agriculture | 4 | 1 |
| D | Estero Arenas | 0.301 | *A. balios* | 2 | 2 |  | Agriculture-cattle | 2 | - |

* Bd= *Batrachochytrium dendrobatidis.* ERS= Environmental Risk Surface value from threat model in Ortega-Andrade et al. [13]

Supplementary Material

Supplementary Material SM1. A standardized protocol to isolate eDNA (water samples), DNA (SWABs), and amplifying PCR products to detect BD with portable devices.

Supplementary Material SM2. Sequences (.fasta) of positive Bd validated in BLASTn from SWABs (2395, 2397, 2420, 2421) and water (1a-16a) samples in the study area. See Table S1 for specimens, access numbers in Genbank and water sample codes.

Supplementary Material SM3. Raw Data for Physical-Chemical water analysis in Figure 3. Raw data of physical-chemical parameters used to generate Figure 3, to analyze the environmental variation and clustering of sampled localities resulting from Principal Component Analysis.

Supplementary Tables.

Table S1. Locations for sampling SWABs on *Atelopus* specimens and water from streams in the study area (Fig. 1). Detailed data on codes, access numbers to Genbank, GPS coordinates, altitude, temperature, and relative humidity (RH) are provided.

Table S2. Methods of analysis and detection limits for physical chemical variables in four sites for assessing the presence of BD in *Atelopus* populations in southern Ecuador.

Table S3. Amphibians sampled with detected Bd in Ecuador.

Supplementary Figures.

S1 Fig. Electrophoresis result for field and laboratory analysis. (A) field laboratory (B) university laboratory.
