# Supplementary material for: Field-based molecular detection of Batrachochytrium dendrobatidis in critically endangered Atelopus toads and aquatic habitats in Ecuador
Source: PLoS One. 2024 Mar 14;19(3):e0299246. doi: 10.1371/journal.pone.0299246 (PMC10939218; doi:10.1371/journal.pone.0299246)
Supplement: Attachment — Submitted filename: Response Reviewers R2.docx [file pone.0299246.s008.docx]

Dear Editor,

We finally have a reviewed version of our manuscript “Field-Based molecular detection of *Batrachochytrium dendrobatidis* in critically endangered *Atelopus* toads and aquatic habitats in Ecuador -PONE-D-23-11480R1) submitted to PloSOne.

Our responses to reviewers are codified as AR.

Review Comments to the Author

Please use the space provided to explain your answers to the questions above. You may also include additional comments for the author, including concerns about dual publication, research ethics, or publication ethics. (Please upload your review as an attachment if it exceeds 20,000 characters)

Reviewer #1: The new version of the document took into consideration the comments from the first revision, generally in a satisfactory manner. The main problem was the title that is not in accordance with the objectives and results presented. In the new version, although some aspects were covered, the conclusions remain unclear what the achievements were.

AR: We appreciate your feedback. We have accordingly updated the text. The conclusions as well as other parts of the text have been completely modified.

Reviewer #3: Dear authors,

The manuscript has been considerably improved and it is now written in a more intelligible way. There are still some minor issues I would recommend you addressing before publication.

I strongly believe you should remove all the information related to physical-chemical analysis of water samples (including figure 3 and supplementary material), as it is not related with the aim of this study, and there is not a clear explanation of the relevance of the findings. Maybe dig a bit more in this in another publication. Also, it would be great if you can discuss the sensitivity of your protocol for Bd detection in water samples.

AR: Thank you for your input. Based on your suggestions, we decided to exclude the details concerning the physico-chemical parameters because consider this results are not directly related with the research question.

Please be consistent within the manuscript. Choose one term (e.g Bd vs chytrid pathogens; chytridiomycosis vs chytridium, in situ lab vs field lab, etc), and stick with it through the text. Otherwise it causes confusion. The same occurs with the time of samples analysis in situ. In the results section you say it takes 6-8 hours, and in the discussion you say it takes 4-6 hours.

AR: We have standardized the terminology used throughout our work. Additionally, we've provided more comprehensive explanations for specific terms and refined the description of the time intervals between sample transportation and extraction, clarifying the significance of these durations.

Some information in the introduction, methods and discussion still need to be rephrased (see below).

Figures 1 and 2 are still blurry. There are still some inconsistencies between the names within figure 1 and the legend. In figure 2, I see some discrepancies between the results obtained in situ and in campus (samples 3a and 4a). See related comments and suggestions below.

AC: The figures were changed; the inconsistencies were corrected.

Line 51: change “Bd in native populations…” to “Bd in wild populations… endangered native species”.

AR: The introduction has been revised, and the relevant section has been updated accordingly.

Line 56: you did not detect Bd prevalence. Please change to: “Bd was detected in…”

AR: The text has been corrected.

Line 60: monitoring of what? You need to say Bd detection (in situ Bd detection).

AR: This was corrected and re-worked. We substitute the in situ for field detection.

Line 61: please change “monitoring of a tropical invasive and zoosporic fungus” to “Bd”. It is better to keep it simple.

AR: The text has been modified.

Line 72: change “mortal registered disease” to “mortal disease reported to date”. Change “produced” for “caused”. Also, Bd IS NOT a zoonotic fungus. Please change “zoonotic” for “chytrid”.

AR: We redo these lines.

Line 74: change “After the” for “After a”.

AR: The text has been corrected for “a” instead of “the”

Lines 78-83: please rephrase, it is confusing.

AR: The text has been rephrase.

Line 81: change “chytridium” for chytridiomycosis. Please be consistent through the manuscript. Use Bd when talking about the pathogen, and chytridiomycosis when talking about the disease.

AR: The text has been corrected and terminology has been checked

Line 91: add “amphibian” after “threatened”.

AR: The text has added

Line 97: change “Ecuador” for “the country”.

The text has been changed

Lines 97-108: please provide similar information about the three species (distribution, Bd status). You say A. nanay lives only in the Cajas National Park, but you don´t say where the other 2 species are found. There is no information concerning Bd in A. balios.

AR: A more detailed description was added in the Study area section

Line 112: the mechanism of what? Do you mean the mechanism of infection? Please add as necessary.

AR: The paragraph has been rewritten

Line 117: change “biomonitoring” for “pathogen surveillance” or “Bd surveillance”.

AR: The suggestion has been implemented

Line 121: change “detect in situ” for “detect the presence of Bd in situ in native…”

AR: The text has been modified. For instance in situ was modified by field laboratory

Line 128: change “security” for “biosafety”. This info should be in the sampling section.

AR: The text has transfer to the sampling section and the text has been rewritted

Line 158: change “were” to “was”.

AR: The suggestion to replace "was" with "were" has been followed.

Lines 158-159: rephrase the swabbing method and cite.

AR: The text has been corrected and cited

Line 163: rephrase the first sentence.

AR: The first sentence was rephrase

Line164: change “arrival” for “arriving”, and “start” for “starting”.

AR: The text has been corrected

Lines 172-181: this information differs from the protocol detailed in SM1. Also, you need to say if the first half of the filter was stored at room temperature, 4°C or other.

AR: The information has been updated. The protocols provided in SM1 has been corrected.

Lines 197-180: change “At the in situ…” to “To prevent contamination at the in situ laboratory, …”.

AR: The changed has been applied “in situ” was substitute with “ in field” .

Line 200: change “Additional” to “ In addition,”.

AR: Agreed and corrected.

Lines 262-264: the information concerning prevalence should be at the end of the previous section.

AR: Corrected.

Line 263: change “is the number” for “ was calculated considering the number…”.

AR: Agreed and corrected.

Lines 275-278: please rephrase, it is confusing.

AR: The entire paragraph has been rephrased.

Line 282: change “this” to “these”.

AR: Agreed and corrected.

Line 286: change "show” to “showed”.

AR: Agreed and corrected.

Line 293: change "ratesthan” to “rates than”

AR: Agreed and corrected.

Line 296: you say you collected one water sample, but Table 1 says you collected wo samples and one was positive.

AR: Inconsistencies were corrected

Line 329: change “with detected BD” to “ positive to Bd”.

AR: Agreed and corrected.

Lines 331-332: The first sentence needs to be rephrased. It is confusing.

AR: We redo part of the discussion section for instance this part was merged with other sections in discussion

Line 334: change “pest management” to “disease surveillance”.

AR: See previous comment.

Line 335: delete “the amphibian”. Delete “epidemic for chytridiomycosis”. Add “in Ecuador” after “in situ”.

AR: We redo part of the discussion section for instance this part was merged with other sections in discussion

Line 338: delete “portable”.

AR: See previous comment.

Line 339: add “portable” after “and”. It should be “laboratories and portable molecular tools…”.

AR: See previous comment.

Line 343: were water samples results the same or similar? I thought they were identical, but after seeing figure 2 and the information presented in line 343, it appears not to be like that.

AR: The water samples for field lab and university lab showed similar results, and these findings were corroborated by subsequent PCR reactions and sequencing.

AR: The discussion here implies that, at some sites, there was no amplification for Bd, even though some species collected nearby tested positive.

Line 347: change “PCR reaction” to “PCR inhibition”.

AR: Agreed and corrected.

Line 348: add “sampled for this study” after “Coastal sites”.

AR: Agreed and corrected.

Line: 349: change “pressures” to “activities”.

AR: The information was rephrased

*I would delete the information presented in lines 348-356. You don´t have enough data to make thi*I would delete the information presented in lines 348-356. s conclusions and it is not essential for this particular study.

Line 357: change “chytrid pathogens” for “Bd”. You are not studying other chytrid fungi.

AR: Agreed and corrected.

Lines 360-370: delete this information. It is all true and interesting, but it is beyond the scope of this study.

AR: The information was deleted.

Lines 376-378: Rephrase, it is confusing.

AR: The lines have been rephrased.

Lines 419-420: cite.

AR: A citation was provided

Lines 420-422: are you sure you want to say this? Maybe you can change “control the prevalence of Bd” to “control the impact of Bd”.

AR: Agreed and corrected.

Line 424: change “sampling campaigns” to “ pathogen or disease surveillance”.

AR: Agreed and corrected.

Line 430: change “of lethal” to “of a lethal”. Delete “and biological invasions”.

AR: Agreed and corrected.

Line 438: change “Our main conclusions are:” to “Our study shows”.

* I would change conclusion (ii). The most important thing here is not the percentage of water samples that tested positive, but to mention and discuss (within the discussion section) the sensitivity of your protocol to detect Bd DNA in water samples.

AR: The conclusion was redo.

SM1: revisar que los protocolos coincidan con la información descrita en métodos. Ej: en el Protocolo de filtración de muestras de agua dice: “Colectar 3 litros de agua”, mientras que en métodos dice: “One liter of water was taken for each site (about 350ml per point)”.

AR:The method section was redo, mistake was due to cross between protocols in different MS

- 3.PROTOCOLO DE EXTRACCIÓN DE ADN METAGENÓMICO DE LOS FILTROS DE AGUA. - Cambiar ADN metagenómico por ADN ambiental.

AR:The title was changed.

- Dice: “cada filtro será cortado en 3 partes”, mientras que en los métodos dice: “filters were divided in two halves”.

The same occurs in the English version.

AR: The error was corrected

Reviewer #4: This manuscript is much improved, but still requires some editing for grammar and clarity. Additionally, I hope the quality of the figures can be improved because they are a bit hard to decipher, especially the text in the red box in Figure 1. I was not able to access the file listed as the supplement because of the unusual file type. Can this be provided as a .zip instead?

Thanks for your comments we have adapted your suggestion to the MS.

Here are a few line comments, but I believe the authors should do one more edit for grammar before resubmitting.

Line 71: “most infectious” and “most mortal” are not well-supported claims. I suggest tempering this language.

AR: The suggestion has been followed.

Line 81: “Chytridium” should be “Chytridiomycosis” and change “being Atelopus toads should be “and Atelopus are one of the most…”

AR: The paragraph was edited

Line 200 – Can you be more specific about which practices were carried out?

AR: The paragraph was redo however this specific practices are now detailed in the MS and in the attachment section.

Line 343 – This sentence makes it sound like the eDNA results were different between the field and university labs, but the results said the results were identical. This is confusing.

AR: We redo the sentence. Here we referred to the no amplification of DNA from specific sites which can be due the physical chemical variables or influenced by human activities.

Line 376 – Change this sentence to “Early detection of invasive or pathogenic species in the field with molecular accuracy is key for monitoring and surveillance for biodiversity conservation.”

AR: The phrase was changed

Table 1 should also include the results from the on-campus lab for the eDNA samples. This can help clear up the confusion from Line 343.

AR: Table 1 was changed
